# The value of radiographic markers in the diagnostic work-up of rotator cuff tears, an arthroscopic correlated study

**DOI:** 10.1007/s00256-019-03251-8

**Published:** 2019-06-14

**Authors:** Jeroen J. van der Reijden, Syert L. Nienhuis, Matthijs P. Somford, Michel P. J. van den Bekerom, Job N. Doornberg, Esther van ‘t Riet, Maaike P. J. van den Borne

**Affiliations:** 1 Department of Radiology, Deventer Ziekenhuis, Nico Bolkesteinlaan 75, 7416 SE Deventer, The Netherlands; 2 Department of Radiology, UMCG, Hanzeplein 1, 9713 GZ Groningen, The Netherlands; 3 Department of Orthopaedic Surgery, Rijnstate Ziekenhuis, Arnhem, The Netherlands; 4 Department of Orthopaedic Surgery, OLVG Amsterdam, Amsterdam, The Netherlands; 5 Department of Orthotrauma Research Center Amsterdam, Amsterdam University Medical Center, location AMC, Amsterdam, The Netherlands; 6 Deventer Ziekenhuis, Deventer, The Netherlands; 7 Department of Orthopaedic Surgery, Amphia ziekenhuis, Breda, The Netherlands

**Keywords:** Rotator cuff tear, Radiographic markers, Diagnostic test, Predictive value, Arthroscopy, Shoulder pathology

## Abstract

**Objective:**

To evaluate the value of radiographs during the diagnostic work-up of rotator cuff tears, using arthroscopy as reference standard.

**Materials and methods:**

This retrospective study included 236 shoulders of 236 patients. All radiographs were evaluated for inferior cortical acromial sclerosis, lateral acromial spur, superior migration of the humeral head, greater tubercle cysts, and subacromial space calcifications. Predictive value of these radiographic signs in predicting rotator cuff tears was determined with arthroscopy as reference standard.

**Results:**

According to arthroscopy, 131 shoulders were diagnosed with rotator cuff tears. Seventy-two out of 131 shoulders (55%) had inferior cortical acromial sclerosis, 37 (28%) lateral acromial spur, 21 (16%) superior migration of the humeral head, 7 (5%) greater tubercle cysts and 15 subacromial space calcifications (11%). Inferior cortical acromial sclerosis (*P* = 0.001), lateral spur (P = 0.001), superior migration (*P* = 0.002), and cysts (*P* = 0.03) were significantly and independently associated with rotator cuff tears, whereas subacromial calcifications (*p* = 0.21) was not. Inferior cortical acromial sclerosis, superior migration, lateral acromial spur, and cysts combined have a positive predictive value of 78%.

**Conclusions:**

The combination of inferior cortical acromial sclerosis, lateral acromial spur, superior migration of the humeral head, and greater tubercle cysts has a high positive predictive value for the presence of full-thickness rotator cuff tears. In patients with a high suspicion for having a rotator cuff tear based on radiographic findings, MRI can be performed directly without the delay and costs caused by an additional ultrasound exam.

## Introduction

**Fig. 1:**
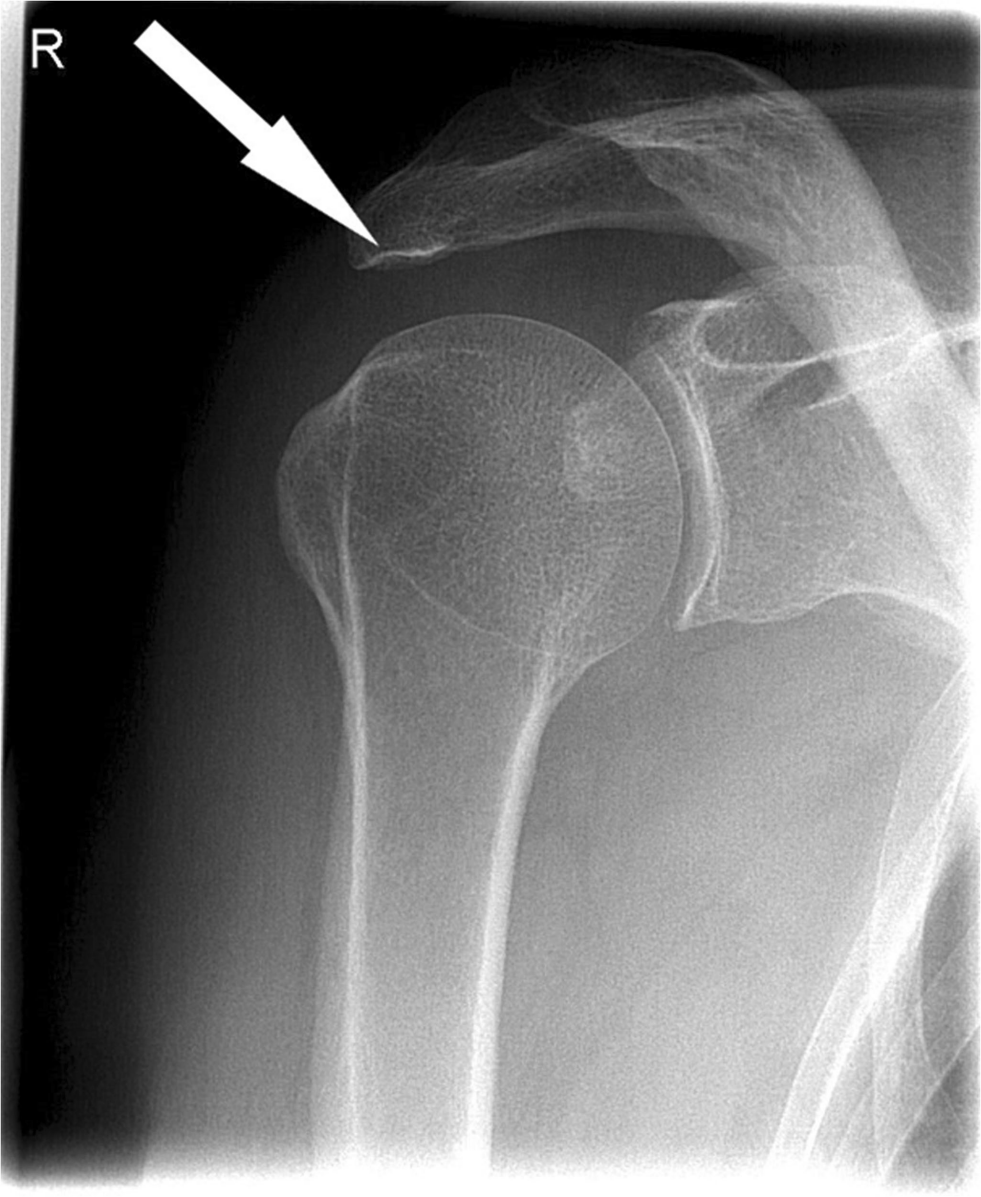
Anteroposterior (AP) conventional radiographs of the shoulder demonstrating several markers. The right shoulder demonstrates inferior cortical acromial sclerosis, i.e., the sourcil sign

**Fig. 2:**
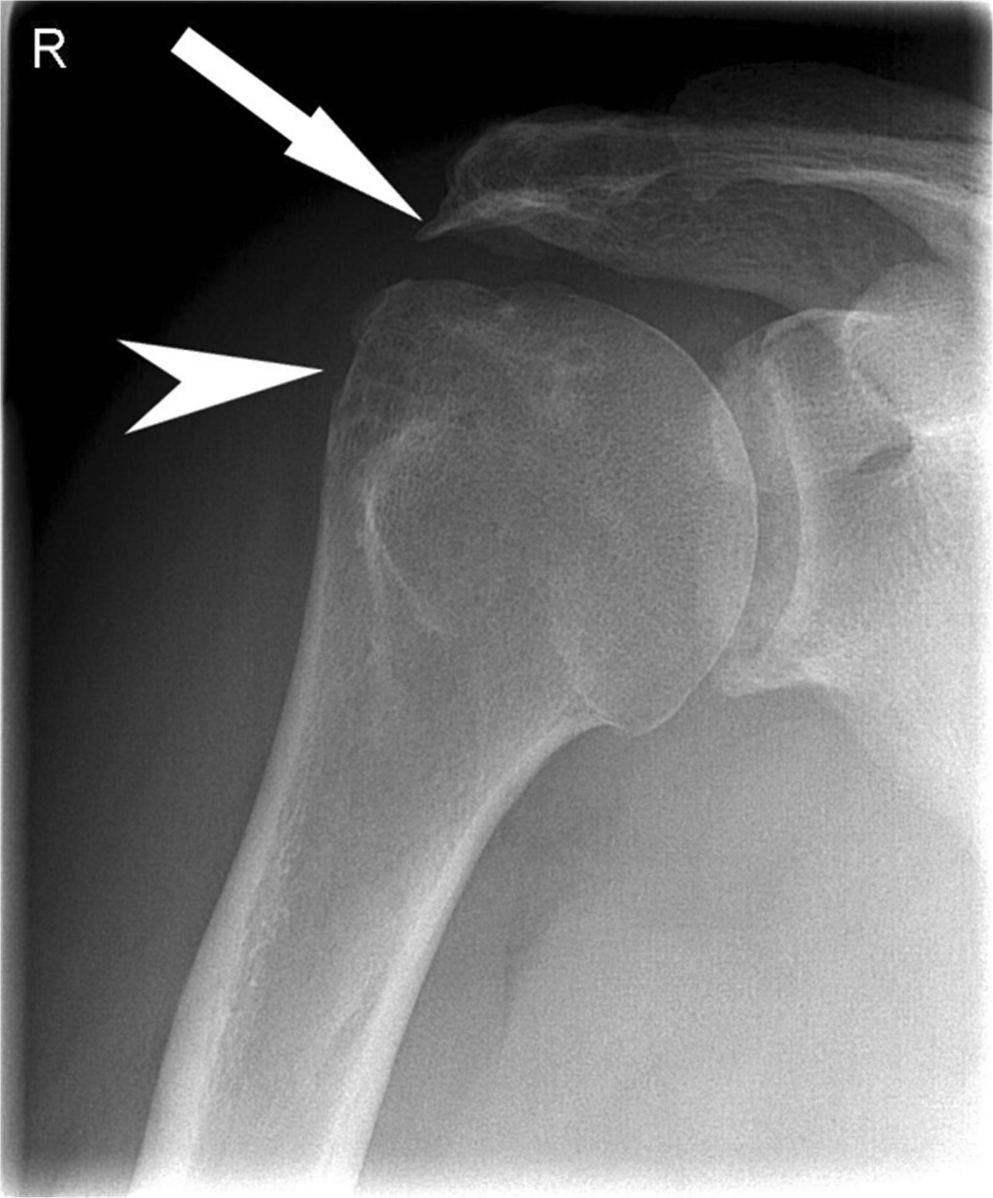
Anteroposterior (AP) conventional radiographs of the shoulder demonstrating several markers. A lateral acromial spur (*straight arrow*) and cysts in the greater tubercle (*arrowhead*) of the right shoulder is shown

**Fig. 3:**
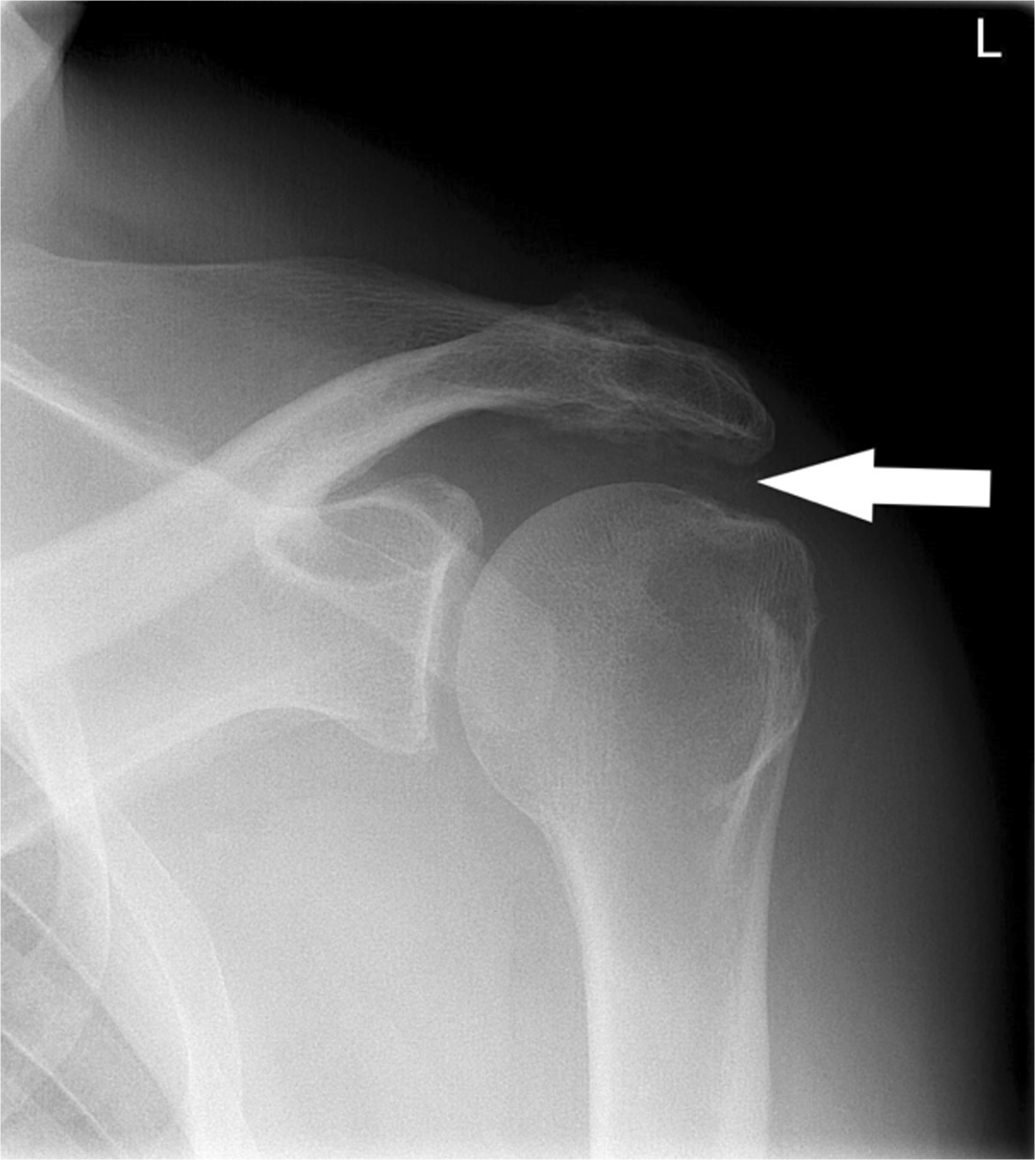
Anteroposterior (AP) conventional radiographs of the shoulder demonstrating several markers. The left shoulder demonstrates calcifications of type C: Non-homogenous calcifications with serrated or irregular contours in the subacromial space are observed

**Fig. 4:**
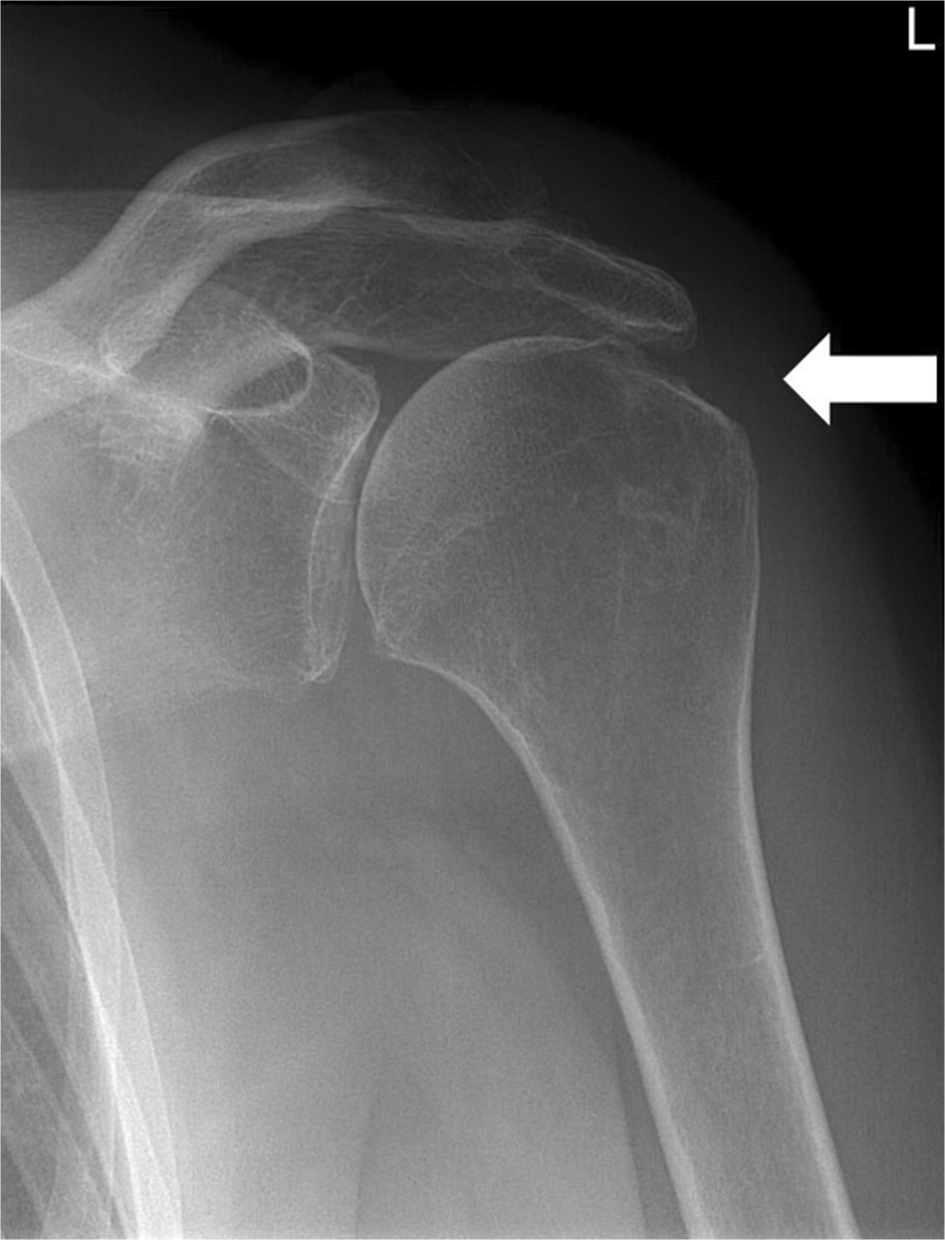
Anteroposterior (AP) conventional radiographs of the shoulder demonstrating several markers. The left shoulder demonstrates superior migration of the humeral head

Many shoulder complaints are caused by rotator cuff pathology such as tendinopathy or rotator cuff (RC) tears. Diagnosis starts with medical history and physical examination, after which radiography is usually the first chosen imaging modality. Radiographs of the shoulder easily show signs of fractures, osteoarthritis, malignancies, and calcific tendinitis [1, 2]. Unfortunately, the full capability of conventional radiographs in establishing the presence of an RC tear is not standardly being used. Various radiographic markers and techniques have been reported to have a predictive value for the presence of RC tears. These include the acromial morphology, inferior cortical acromial sclerosis (also known as the "sourcil sign"), acromial spurs, acromioclavicular joint osteoarthritis, superior migration of the humeral head, calcifications in the rotator cuff, and degenerative changes of the glenoid or humeral head such as sclerosis or irregularity of the greater tuberosity, greater tuberosity cysts, and osteophytes [1, 3–11]. Previous studies [2, 10–15] showed correlation of these markers and the presence of RC tears. Those studies had a relatively small population, analyzed only one or a few markers, or were not arthroscopically assessed. Therefore, the true value of the radiographic signs is still unclear. The purpose of this study is to evaluate the predictive value of radiographic signs for the presence of RC tears during the initial diagnostic work-up, using shoulder arthroscopy as the reference standard. The hypothesis of our study is that radiographic markers such as inferior cortical acromial sclerosis, a lateral acromial spur, superior migration of the humeral head, cysts in the greater tubercle and calcifications in the cuff are correlated with the presence of a RC tear, as diagnosed during arthroscopy (Figs. 1, 2, 3, and 4). If specific markers are proven to correlate with the presence of a rotator cuff tear, the orthopedic surgeons’ suspicion will be validated and give direction to complementary tests. Both ultrasound and MRI were performed in multiple hospitals as standard work-up in rotator cuff tears. However, in patients with a high suspicion of a RC tear, only one additional diagnostic test could be sufficient as a basis of whether or not to perform surgery. Therefore, our aim is to prove a high positive predictive value (PPV) of markers on radiographs for the presence of RC tears. With a high PPV, a radiograph could be of diagnostic value in the work-up of RC tears and give direction for the additional diagnostic test needed.

## Materials and methods

### Study design

In this retrospective study, 236 shoulders of 236 consecutive patients who underwent a shoulder arthroscopy between 2010 and 2012 were selected. Only patients with pre-operative AP radiographs of the affected shoulder in internal- and external rotation were included. Patients with previous surgery or inflammatory diseases of the shoulder were excluded. Two experienced orthopedic surgeons assessed sets of pre-operative radiographs of the affected shoulders in consensus. The patients were divided into three groups. The first group consisted of 111 patients (111 shoulders) with full-thickness rotator cuff (FTRC) tears of the supraspinatus, infraspinatus, or the subscapularis (64 male, mean age 61 years; 47 female, mean age 60 years). The second group consisted of 20 patients (20 shoulders) with partial-thickness rotator cuff tears (PTRC tears) (ten male, mean age 53 years; ten female, mean age 60 years). The third group consisted of 105 patients (105 shoulders) with an intact rotator cuff (54 male, mean age 46 years; 51 female, mean age 47 years). See Table 1 for sex and age demographics. Ethical approval: for this retrospective study, formal consent was not required according to the ethical standards.

**Table 1:** Ratio of total men and women and their age per tear type

	FTRC tear	%	PTRC tear	%	No RC tear	%	Total
All patients	111		20		105		236
Men	64	50	10	8	54	42	128
Mean age, men (years)	61		53		46		53
Women	47	44	10	9	51	47	108
Mean age, women (years)	60		60		47		56

*RC tear* rotator cuff tear, *FTRC tear* full-thickness rotator cuff tear, *PTRC tear* partial-thickness rotator cuff tear

### Radiographic evaluation

Radiographs were anonymized in accordance with terms of privacy. All radiographs were converted to a DICOM file. One case was created for each patient that was randomly numbered. The following items were assessed in consensus by two experienced orthopedic surgeons using an online platform: “Presence of inferior cortical acromial sclerosis?” (representing the sourcil sign, defined as a hyperdense delineated undersurface of the acromion), “Presence of a lateral acromial spur?” (any bony extension along the lateral side of the acromion as seen on AP radiographs), “Is there superior migration of the humeral head?” (glenohumeral relation was distorted by any form of superior migration), “Presence of cyst(s) in the greater tuberosity?” (lucencies with a sclerotic edge present in the greater tubercle) and “Calcifications in the rotator cuff trajectory using Mole’s classification (1993) as stated in the ISAKOKS/ESSKA standard terminology, definitions, classification, and scoring systems for arthroscopy” [16]. All the questions were dichotomous (yes/no) except for the fifth question, which was multiple choice (“Type A: Dense homogenous calcifications with clear contours.”, “Type B: Dense split or separated calcifications with clear contours.”, “Type C: Non-homogenous calcifications with serrated or irregular contours.”, “Type D: Dystrophic calcifications of the insertion in continuity with the tuberosity.” and “Type E: None”). The researchers in consensus assessed all radiographs and were blinded for the symptoms of the patient, the outcome of the arthroscopy, and age and gender of the patients. For each marker in each group, the prevalence, sensitivity, specificity, positive predictive value (PPV), negative predictive value (NPV), and odds ratio were analyzed.

### Arthroscopy

Two experienced orthopedic surgeons performed the arthroscopies. When a complete rupture in one of the rotator cuff tendons was diagnosed during arthroscopy, with any form of retraction and also appeared completely loose of the original site of attachment, this was defined as an FTRC tear. When the rupture was only partially through the tendon, there was no retraction and the rest of the tendon appeared stable, this was defined as a partial PTRC tear. The definition of an intact rotator cuff was when the rotator cuff appeared completely intact during arthroscopy.

### Statistical analysis

Statistical analysis was performed with SPSS statistical software version 23 (SPSS Inc., Chicago, IL, USA). Two groups were created, the first group with a RC tear and a control group with an intact rotator cuff. A second group was created with an FTRC tear and the same control group. These two groups were analyzed for correlations of all different variables and whether or not these variables were significant independent predictors for the presence of a RC tear. Statistical significance was defined as a *P* value < 0.05. Thereafter, a logistic regression model was used as a model to study whether a combination of variables can attribute to a superior prediction for the presence of an RC tear. The presence of cysts was excluded for this analysis because of the specificity of 100% and it could therefore not be included in the logistic regression model. However, cysts were included in the individual prediction of each patient. Hereupon, a manual backward elimination logistic regression was performed. Variables were eliminated from the model if the relationship between that variable and the outcome had a significance level of *P* > 0.10. In addition, by comparing the −2 log likelihood after each removal of a variable, the performance of the model was indicated. To assess the goodness of fit of the final model, the Hosmer and Lemeshow test was performed. In addition, the individual prediction of each patient in the dataset was calculated using the regression function of the final regression model. The presence of cysts was entered into the individual prediction manually (the presence of cysts was translated into a 100% prediction). The individual prediction was used to estimate the area under the curve (AUC) of the receiver-operating curve (ROC), which was calculated as a measure of predictive ability of the model. Then, the most optimal scale limit as seen on the ROC curve was chosen, defined as the individual prediction level with the PPV. All cases were arranged as above or below this cut-off value and were then correlated to the actual presence or absence of a RC tear. A statistical analysis of the PTRC tear group was not performed, because of an insufficient number of patients for a correct analysis using a multivariable logistic regression analysis.

## Results

### Radiographic evaluation

A total of 236 patients/shoulders were included in this study. There were no significant differences in sex and age between the groups. Ninety-three shoulders (39%) had inferior cortical acromial sclerosis, 39 shoulders (17%) a lateral acromial spur, 26 shoulders (11%) superior migration of the humeral head, seven shoulders (3%) cysts in the greater tubercle, 38 shoulders (16%) any calcification, 11 (3%) calcification type A, 5 (13%) calcification type B, 17 (45%) type C, and 5 (13%) calcification type D (Table 2).

**Table 2:** Variety of total radiographic markers on radiographs in correlation with intact or tears of the rotator cuff

	All shoulders	No tear	RC tear	*P* value	FTRC tear	*P* value
*N* (%)	236	105 (44%)	131 (56%)		111 (47%)	
Inferior cortical acromial sclerosis	93 (39%)	21 (20%)	72 (55%)	< 0.001	62 (56%)	< 0.001
Lateral acromial spur	39 (17%)	2 (2%)	37 (28%)	< 0.001	31 (28%)	< 0.001
Superior migration humeral head	26 (11%)	5 (5%)	21 (16%)	0.006	20 (18%)	0.002
Cysts greater tubercle	7 (3%)	0 (0%)	7 (5%)	0.02	6 (5%)	0.03
Calcifications						
None	198 (84%)	82 (78%)	116 (89%)	0.16	98 (88%)	0.21
Type A	11 (29%)	6 (6%)	5 (4%)		4 (4%)	
Type B	5 (13%)	4 (4%)	1 (1%)		1 (1%)	
Type C	17 (45%)	11 (10%)	6 (5%)		5 (5%)	
Type D	5 (13%)	2 (2%)	3(2%)		3 (3%)	

*RC tear* rotator cuff tear, *FTRC tear* full-thickness rotator cuff tear

### Arthroscopy

According to arthroscopy, 105 shoulders (44%) had no RC tear, 20 shoulders (8%) had a PTRC tear, and 111 shoulders (47%) had an FTRC tear (Table 2).

### Statistical results

In multivariate logistic regression analysis, inferior cortical acromial sclerosis, lateral acromial spur, superior migration of the humeral head, cysts in the greater tubercle, and cuff tear suspicion were independently significant (*p* < 0.05). Subacromial calcifications were not significant (Table 2). Then a manual backward elimination logistic regression was performed. In the RC tear group, the optimal model was achieved when combining inferior cortical acromial sclerosis and a lateral acromial spur. In the FTRC group, the optimal model was achieved when combining inferior cortical acromial sclerosis, lateral acromial spur, and superior migration. Our definite regression equation for the predicted possibility of the presence of a RC tear was: − 0.395 + 1.059 × inferior cortical acromial sclerosis + 2.38 × lateral acromial spur. The definite regression equation for the FTRC tear group was − 0.634 + 0.979 × inferior cortical acromial sclerosis + 2339 × lateral acromial spur + 0.995 × superior migration. The Hosmer and Lemenshow test for both models was not significant, indicating a good fit of the model. Adding the variable cysts to the individual prediction completed the individual prediction resulting from these models. The AUC of the ROC curve was calculated for the individual prediction with and without the manual addition of the variable cysts. Both AUCs were 0.71 in the RC group and 0.72 in the FTRC group. The optimal cut-off value according to ROC analysis yielded a PPV of 78% in the RC tear group (with sensitivity, specificity, NPV, and odds ratio respectively of 57%, 80%, 60% and 5.40) and 74% in the FTRC tear group (with sensitivity, specificity, NPV, and odds ratio, respectively, of 60%, 77%, 65% and 5.02) (Table 3).

**Table 3:** Diagnostic characteristics of the RC tear and FTRC tear model vs. no tear

Characteristic	RC tear group	FTRC tear group
Sensitivity	57%	60%
Specificity	80%	77%
Positive predictive value	78%	74%
Negative predictive value	60%	65%
Diagnostic odds ratio	5.40	5.02

Rotator cuff (RC) tear group model consists of the combination of inferior cortical acromial sclerosis, lateral acromial spur and greater tubercle cysts

Full-thickness rotator cuff (FTRC) tear group model consists of the combination of inferior cortical acromial sclerosis, lateral acromial spur, superior migration, and greater tubercle cysts

## Discussion

The results of this study show that inferior cortical acromial sclerosis, a lateral acromial spur, the presence of cysts in the greater tubercle of the humerus or superior migration of the humeral head, can accurately be used as radiographic markers to raise the suspicion of a RC tear. The highest PPV for the presence of a RC tear is the combination of inferior cortical acromial sclerosis, a lateral acromial spur and cysts in the greater tubercle (78%). The highest PPV for the presence of an FTRC tear is the combination of inferior cortical acromial sclerosis, a lateral acromial spur, superior migration of the humeral head, and cysts in the greater tubercle (74%). As hypothesized, certain markers are closely correlated with the presence of RC tears. After taking the history and physical examination at the outpatient clinic, a conventional radiograph of the affected shoulder is performed. In multiple hospitals, the first step in the diagnostic work-up of RC tears is ultrasonography. In case of an RC tear, an MRI will be performed to assess the characteristics of the tear, visualize retraction, atrophy or fatty degenerative changes. In some centers and/or situations, the decision to perform surgery is based solely on ultrasound. In other hospitals, only one additional diagnostic modality is selected (ultrasound or MRI).

However, the level of suspicion could be raised after a radiograph with a high PPV for RC tears. A high level of suspicion, based on the radiograph, results in the need of only one complementary diagnostic test (usually MRI) to confirm the diagnosis together with starting conservative treatment or planning and performing surgery. This results in less time between the patients’ first visit and start of treatment. Since an MRI shows more tear characteristics, retraction and fatty degeneration of a tear, an additional ultrasound should be avoided when a high suspicion of an RC tear is present based on the initial work-up of physical examination and a radiograph. This would be both time- and cost-effective. Our study showed a positive correlation of inferior cortical acromial sclerosis, lateral acromial spur, superior migration, and cysts with RC tears. A large variety of radiographic markers were analyzed in our study and were in line with previous studies [3, 7, 9, 13–15, 17–19]. However, other studies described no correlation between cysts and lateral acromial spur on radiograph and RC tears [5, 20–22]. Umans et al. [14] used arthroscopy as the gold standard when investigating the correlation between radiographic markers and RC tears in a small group of 40 patients. Ghandour et al. had analyzed a large cohort of 425 patients for only greater tuberosity sclerosis, but not all of them underwent arthroscopy [11]. The strength of our study is a large variety and a combination of these radiographic markers for RC tears in a large cohort with arthroscopy as gold standard. The combination of our markers has a higher PPV then Hussain et al. reported in a large study when both cortical irregularity and sclerosis is present [10]. The online database is easily accessible and minimizes inter-software discrepancies. However, only anterior-posterior radiographs were assessed and the inability to perform measurements is limiting the extent of markers that could be analyzed (like critical shoulder angle and acromion shape). Another limitation is that our study does not distinguish between traumatic and non-traumatic-induced RC tears. Recent research indicated that degenerative RC tears are associated with a narrower subacromial space, a larger lateral acromial spur, as well as a steeper angulation of the acromion in respect to traumatic tears [23]. This might imply more accurate results in a patient group with chronic shoulder complaints. The selection of our patient group might be biased, as they all had undergone arthroscopy because of symptomatic complaints of their shoulder. In our study, all patients were symptomatic, so there was not an optimal control group. However, adolescents are also included in this study where no degeneration is to be expected. Furthermore, studying shoulder radiographs in asymptomatic patients is less relevant for this study since that group will not attend an outpatient clinic and the underlying cause of shoulder complaints vary per patient. To minimize bias, the radiographs were anonymized for age and sex. However, large age differences can be noticed on a radiograph, which may have biased the interpretation, because for a young adult it is less likely to have an RC tear. One of the observers also performed arthroscopy and in theory, due to the retrospective setup of this study, might have recognized pathology of the radiographs. In our study, solitary subscapularis tears were not excluded because a legitimate patient group was simulated. An orthopedic surgeon is not able to differentiate between rotator cuff tear types (for instance partial full-thickness or partial-thickness tears) beforehand. When excluded, we would create a selection of patients that is not representative of the clinical setting. Unfortunately, there were not enough patients having a PTRC tear to correctly perform a logistic regression analysis. Finally, in this study, two observers assessed the radiographs in consensus. Using this set-up could account for inter-observer variability. Even though we only used two observers, already meaningful results were obtained. Also, because disagreement was solved by consensus, the inter-observer reliability was not calculated, being a limitation. Furthermore, the fact that radiographs were not analyzed by radiologists is a limitation. However to validate the role of a radiograph in the work-up of RC tears, more research with more observers is needed. Studies with measurements within radiographs, radiologists as observers, large cohorts, the correlation of radiographic markers with PTRC tears and less selection bias should be investigated. The variety of radiographic markers should not only be assessed by arthroscopy but also evaluated with MRI and ultrasound to validate the suspicion of an RC tear. More randomized studies should be performed to study the value of radiographs in the diagnostic work-up of RC tears in centers where ultrasound is performed as first diagnostic imaging modality when being followed by MRI to assess whether or not to perform surgical treatment.

In conclusion, the presence of inferior cortical acromial sclerosis, a lateral acromial spur, superior migration of the humeral head, and cysts in the greater tubercle are all legitimate and independent significant predictors for the presence of an RC tear. The combination of these predictors provides a high PPV for having an FTRC tear. Eventually, with a high probability of an RC tear based on conventional radiographs, only one additional diagnostic test should be sufficient to confirm the diagnosis and base the decision to perform additional treatment. For centers who use both ultrasound and MRI in the diagnostic work-up and decision-making for treating RC tears, only performing MRI is sufficient, which is time- and cost-effective.
